# Development of tools to study personal weight control strategies: OxFAB taxonomy

**DOI:** 10.1002/oby.21341

**Published:** 2016-01-08

**Authors:** Jamie Hartmann‐Boyce, Paul Aveyard, Constantinos Koshiaris, Susan A. Jebb

**Affiliations:** ^1^ Nuffield Department of Primary Care Health SciencesUniversity of OxfordOxfordUK

## Abstract

**Objective:**

To describe the development of the Oxford Food and Activity Behaviors (OxFAB) taxonomy and questionnaire to explore the cognitive and behavioral strategies used by individuals during weight management attempts.

**Methods:**

The taxonomy was constructed through a qualitative analysis of existing resources and a review of existing behavior change taxonomies and theories. The taxonomy was translated into a questionnaire to identify strategies used by individuals. Think‐aloud interviews were conducted to test the face/concept validity of the questionnaire, and test–retest reliability was assessed in a sample of 138 participants.

**Results:**

The OxFAB taxonomy consists of 117 strategies grouped into 23 domains. Compared to taxonomies used to describe interventions, around half of the domains and strategies identified are unique to the OxFAB taxonomy. The OxFAB questionnaire consists of 117 questions, one for each strategy from the taxonomy. Test–retest resulted in a mean PABAK score of 0.61 (SD 0.15). Questions were revised where appropriate.

**Conclusions:**

The OxFAB taxonomy and questionnaire provide a conceptual framework to identify the cognitive and behavioral strategies used by individuals during attempts at weight control.

## Introduction

Excess weight is a major cause of preventable morbidity and mortality, putting individuals at increased risk of conditions such as type 2 diabetes and cardiovascular disease. The World Health Organization estimates that excess weight causes at least 2.8 million deaths and 35.8 million disability adjusted life years annually 1. Obesity arises as a consequence of energy intake exceeding energy expenditure, but attempts to change dietary and activity behaviors to create negative energy balance are often unsuccessful. This is not to say that individuals are not trying to manage their weight: at any one time, around a quarter of American adults are trying to lose weight (27% women, 22% men) 2.

Weight management research has begun to address not only what changes in diet and physical activity are needed, but how these changes are made and maintained. Recently, behavior change interventions targeting obesity have become increasingly complex and have begun to show promise 3, 4. Progress has also been made to classify the specific intervention techniques used in such programs, following guidance on reporting of nonpharmacologic treatments 5, 6. In particular, Michie et al. have created a number of behavior change taxonomies; their most recent taxonomy, designed to apply to a range of behavior change interventions, includes 93 items 7. In addition, taxonomies of behavior change techniques intended for particular issues, including smoking cessation and weight management (most recently, the CALO‐RE taxonomy), have been developed 8, 9. Subject‐specific taxonomies take into account those behaviors which are relevant only to certain behavior change targets (e.g., smoking cessation) and which would be missed if one was coding using a subject‐agnostic taxonomy.

These taxonomies can be instrumental in categorizing intervention components, establishing a common language, identifying active ingredients, and translating effective interventions into practice 7. One limitation, however, is that they have been designed for the purpose of categorizing, designing, and conducting interventions. They adopt an interventionist‐centred approach, classifying behavior change techniques that an interventionist may deliver within a program, rather than the behavioral strategies an individual enacts. In some cases the two approaches may relate closely to each other e.g., goal setting or self monitoring, but many other strategies may be specific to techniques deployed by an individual.

Existing questionnaires designed to explore weight‐related behaviors in individuals are unable to fill this gap, as they primarily focus on quantifying and qualifying intake and expenditure (how much and what kind), e.g., DINE 10 or IPAQ 11, measuring the end result of a myriad of behaviors as opposed to the cognitive and behavioral strategies individuals adopt and which lead to the recorded diet and activity levels 12, 13, 14. The lack of a common framework with which to categorize and evaluate the specific actions taken by individuals for the explicit purpose of weight management hampers the ability of researchers to identify which cognitive and behavioral strategies are deployed and those that may be effective for weight loss and maintenance. The Oxford Food and Activity Behaviors (OxFAB) taxonomy and questionnaire seeks to fill this gap.

## Methods

**Figure 1:**
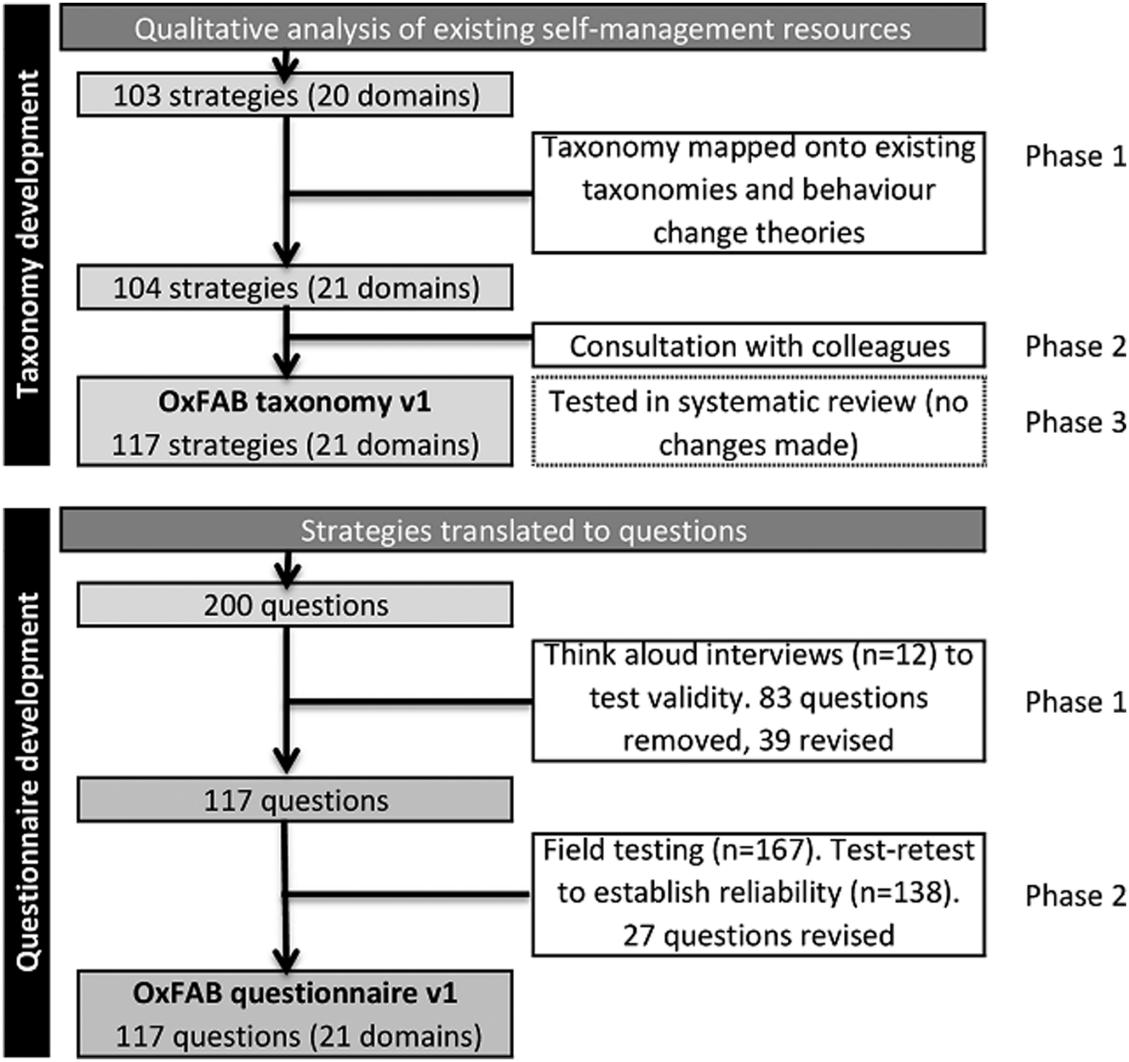
Flow diagram outlining item generation, reduction, and refinement.

Methods for taxonomy and questionnaire development are summarized in Figure 1 and described in more detail below.

### Taxonomy development

The initial stage of development of the OxFAB taxonomy was a three‐phase process. First, a preliminary list of domains relating to weight management was developed, within which there are several strategies which share a common characteristic relating to the domain. Second, the domains and strategies were compared and contrasted with existing taxonomies and theories and refined accordingly. Third, we used the initial version of the taxonomy to categorise interventions in a systematic review of self‐help interventions for weight loss to test and improve ease of use and to identify any further categories. The latter two stages drew on the approach used by Michie and Abraham in their initial development of the Behavior Change Technique Taxonomy 15.

### Building the initial taxonomy

A qualitative analysis of existing weight management resources was conducted to extract individual strategies. We evaluated government resources, representing the standard advice for weight management, and the most popular commercial resources based on UK sales and usage data. Strategies were defined as the cognitive and behavioral techniques adopted by an individual with the aim of improving diet or physical activity for the specific purpose of weight management. Individual strategies were required to be replicable, irreducible (e.g., non‐overlapping and non‐redundant), and mutually exclusive, and we aimed to be as comprehensive as possible within the scope of the current study 7.

Broad categories of techniques, termed domains, were developed drawing on a grounded framework approach 16. Sources were chosen to reflect the most widely used across a variety of formats (Table 1). It was not practicable to seek to be comprehensive in the resources reviewed, but we aimed to ensure key strategies were captured. Reviewing of further resources was stopped when saturation was reached. Individual strategies were extracted verbatim, and using a framework approach, grouped into larger categories to form a hierarchically structured taxonomy. Two distinct forms of categorization emerged: domains were defined as those categories which were the lowest grouping possible above the level of the individual strategy, for example, “self‐monitoring”; and cross classifications were defined as threads that ran through numerous domains, for example “food” or “physical activity.” The framework was translated into a list of domains (e.g., “self‐monitoring”) to which the cross classifications could be applied.

**Table 1:** Self‐management resources used in qualitative framework analysis

Source title	Format	Accessed	Source type	Home page (if relevant)
**Boots/WebMD Diet and Weight Loss Guide**	Website	14 Oct 2013	Commercial	www.webmd.boots.com/diet/guide/diet-weight-loss-losing-weight
**Cancer Research UK, 10 Top Tips**	Print	Published 2011	Charity	n/a
**MyFitnessPal**	App/website	10 Oct 2013	Commercial	www.myfitnesspal.com
**NHS Choices 12‐week guide**	Website	30 Sept 2013	Government	www.nhs.uk/Livewell/weight-loss-guide/Pages/weight-loss-guide.aspx
**Rosemary Conley**	Website	6 Nov 2013	Commercial	www.rosemaryconley.com
**Slimming World**	Website	6 Nov 2013	Commercial	www.slimmingworld.com
**Weight Watchers**	Website	6 Nov 2013	Commercial	www.weightwatchers.co.uk a

a Additional information provided from company as part of previous research project.

Once the initial list had been developed through the grounded framework approach, colleagues in several different departments with expertise in weight management and behavior were consulted to identify additional strategies and domains. Each domain was considered individually and strategies within that domain were brainstormed. Strategies that were not covered were considered by the group of authors and either added to an existing domain or a new domain was created.

### Evaluating the taxonomy in light of existing theory

In phase 2, the overarching strategies were evaluated in the light of existing behavior change taxonomies (at the level of both individual techniques and domains) using a top‐down approach to determine if any groups of strategies had been missed 7, 8. Domains from the OxFAB taxonomy were also mapped onto existing theoretical frameworks of behavior change, including learning theories, social cognitive theory 17, the theory of reasoned action 18, the theory of planned behavior 19, the health action process approach (HAPA) 20, and the PRIME theory of motivation 21. For example, planning elements of the taxonomy were mapped onto the behavioral intention component of the theory of reasoned action, and the preaction stage of the HAPA approach. Having mapped from the domains to the theories, reverse mapping from the theories to the domains was conducted to examine whether any elements had been missed.

### Applying the taxonomy

In phase 3, a simple coding manual was developed, listing the domains, with definitions agreed through discussion, and examples added to aid comprehension. Its use was piloted in a systematic review of randomized controlled trials of self‐directed interventions for weight loss in adults with overweight and obesity 22. Two reviewers (one involved with development of the OxFAB taxonomy and one who was not previously familiar with the taxonomy) independently coded each intervention by domain and, within that domain, by cross classification, as yes, no, or unclear for recommendation of that strategy. Unclear was used where it was agreed the strategy was implied, but not explicitly stated. Table 2 shows an extract of the checklist used. Domains where agreement was low were identified, definitions were adjusted in an iterative process and examples added to produce the working taxonomy.

**Table 2:** Extract from coding checklist

Domain	Definition	Cross classifications			Notes (page #)
		Food	Activity	Other	
**Energy compensation**	Conscious adjustment of behaviors to alter energy intake and/or expenditure in light of previous energy intake or expenditure. Example: If you've eaten a lot, exercise more to make up for it.				
**Imitation (modeling)**	Emulating the physical activity or dieting behavior of someone who you have observed. Example: Choose to go on a certain diet because someone you know lost weight using the same approach.				

### Questionnaire development

Once the working taxonomy was agreed, the 117 strategies were translated into a questionnaire for use by individuals. Initially, multiple questions were developed for single strategies and refined as a result of the pilot testing. Pilot testing was performed in two stages: first, qualitative testing of the questions using the think‐aloud technique (a form of cognitive interviewing) to test face validity, select the optimal question for each strategy and refine the wording to produce a field‐test version; and second, a quantitative Web‐based test–retest study to assess reliability. Both phases of questionnaire development were approved by the University of Oxford Central University Research Ethics Committee and all participants provided informed consent.

### Think aloud (cognitive testing)

Think aloud is a form of cognitive interviewing that has been designed to provide verbal data about reasoning during set tasks 23. Increasingly it is used as a method to establish validity during questionnaire development 24, 25. Participants were recruited from a sample of volunteers drawn from the general public who volunteered for a television weight loss program, and were selected through purposive sampling to ensure representation from men and women, a range of ages, and a range of educational backgrounds. To be eligible, participants were required to be adults with overweight or obesity, resident in the UK, fluent in English, and currently trying to lose weight through changes to their diet, physical activity, or both. Participants were asked to briefly detail the things they were doing to try to lose weight. The interviewer read out each question, and asked participants to answer the question whilst talking through their reasoning. Questions were divided between participants to ensure every question was appraised by three participants. Where there were multiple questions for a single strategy, participants were asked to indicate which they preferred and why. Interviews were audiotaped and transcribed. The transcripts were analysed, with responses coded by question. Where the reasons given for responses were not congruent with the intent of the question, questions were rephrased. Where multiple questions existed for one strategy, the question with participant responses that were most congruent with the question's intent were selected. A final open question enabled participants to share further thoughts on the questionnaire, these were coded and analysed separately, in some instances leading to changes in wording across multiple questions (for example, ‘weight control’ changed to ‘weight management’ throughout).

### Test–retest

After revision in line with think‐aloud responses, the questionnaire was administered online in a multiple choice format including 117 questions about self‐management strategies. Responses included: most of the time; sometimes; never or hardly ever; not relevant to me; and unclear. Participants were recruited through the charity Weight Concerns’ Big Panel, an online panel of people with experience of being overweight (www.weightconcern.org.uk/node/21), and through snowball sampling using people who had participated in previous research. Inclusion criteria were the same as those used for the think‐aloud phase.

The target sample size was 130 participants, based on a sample size calculation of 126 to achieve 80% power to detect a kappa of at least 0.41 (considered moderate agreement) 26. This threshold was chosen as, given the nature of the questionnaire, some genuine changes in behaviors were expected. Participants were asked to repeat the questionnaire 1 to 2 weeks after initial completion. Using data from the first and second testing rounds, the prevalence index, the bias index, and the PABAK (prevalence and bias adjusted kappa) were calculated 27.

Multiple choice answers were coded as ‘yes’ including responses marked as most of the time or sometimes and ‘no’ as never or hardly ever and not relevant to me. Questions where test–retest resulted in PABAK scores lower than 0.41 were re‐evaluated and rephrased as appropriate 28. Using only the first round of participant responses to avoid double counting, questions where more than one participant selected “unclear” were also revisited and rephrased.

## Results

### Taxonomy

One additional domain was added during the process of mapping behavior change taxonomies and theories onto the taxonomy. Prompted by social cognitive theory and learning theories, imitation (modeling) was added. Through discussions with colleagues, 11 additional strategies were also added. Accordingly, the OxFAB taxonomy (version 1) consists of 117 strategies grouped into 23 domains. The list, with definitions and an example strategy can be found in Table 3. The full list of strategies, grouped by domain, can be found in Supporting Information Table S1. The systematic review resulted in minor changes to the coding manual to clarify definitions and examples, but did not uncover previously unidentified strategies, and did not result in substantive changes to the content of the taxonomy. The initial coding yielded a list of 103 strategies.

Compared with existing taxonomies, there was some direct overlap, for example, self‐monitoring 7, 8. Of the 23 domains, 13 included strategies which could not be mapped onto existing taxonomies. When mapped against the most recent, 93‐item behavior change technique taxonomy 7, approximately half of the OxFAB strategies could not be mapped directly on to the behavior change technique taxonomy (see Supporting Information Table S3). Three OxFAB strategies overlapped exactly with techniques from the BCT taxonomy (e.g., “Pledge/agree to contract regarding your weight loss targets” is conceptually the same as BCT 1.8 Behavioral contract), 54 OxFAB strategies provided grounded examples of BCT techniques from the perspective of the participant (e.g., “Measure the amount of physical activity you do” is a grounded example of BCT 2.3 Self‐monitoring of behavior), 23 OxFAB strategies did not map onto any specific techniques but were related to concepts present in the BCT taxonomy (e.g., “Enhance accountability to buddy” is related to BCT category 3, Social support), and 37 OxFAB strategies were not related to concepts itemized in the BCT taxonomy, usually because of their specificity to weight loss (e.g., “Eat slowly.”) The strategies in the OxFAB taxonomy tended to be more detailed and specific than the items in the BCT taxonomy, reflecting the different aims of the two taxonomies.

**Table 3:** Domains of self‐management strategies for weight loss/maintenance

Domain	Definition	Example
**Energy compensation**	Conscious adjustment of behaviors to alter energy intake and/or expenditure to control weight in light of previous energy intake or expenditure	If you've eaten a lot, exercise more to make up for it
**Goal setting**	Setting of specific behavioral or outcome target(s)	Set a goal for how much weight you want to lose by a certain time point
**Imitation (modeling)**	Emulating the physical activity or dieting behavior of someone who you have observed	Choose to go on a certain diet because someone you know lost weight using the same approach
**Impulse management: Acceptance**	Respond to unwanted impulses through awareness and acceptance of the feeling that generates the impulse and reacting without distress or over‐analysis	When you are being physically active and it becomes uncomfortable, accept that it is part of exercising and continue on with your activity
**Impulse management: Awareness of motives**	Respond to unwanted impulses by evaluating personal motives behind that impulse before acting	When you find yourself wanting to eat, ask yourself if you are hungry and only eat if you are
**Impulse management: Distraction**	Respond to unwanted impulses through distraction in an attempt not to act on the impulse	When you feel like eating, distract yourself by doing something else to keep you from eating
**Information seeking**	Seek specific information to enhance knowledge to help manage weight	Look up the calorie content of something you are considering eating using an app or website
**Motivation**	Strategies to increase the desire to control weight	Put a picture of yourself when you were slimmer on your fridge
**Planning content**	Plan types of food/physical activity in advance of performing behavior	Prepare a shopping list in advance of going grocery shopping
**Scheduling of diet and activity**	Plan timing and context/location of food/physical activity in advance of performing behavior	Schedule doing your food shopping at a time when you are unlikely to be hungry
**Regulation: Allowances**	Unrestricted consumption of or access to prespecified foods or behaviors	Allow yourself to eat unlimited amounts of certain foods/drinks
**Regulation: Restrictions**	Avoid or restrict prespecified foods, behaviors, or settings	Never go to fast food restaurants
**Regulation: Rule setting**	Mandate responses to specific situations	Order a small dish when eating out
**Restraint**	Conscious restriction over the amount that is eaten	Accept some periods you will stick to your diet more than you will at other times (flexible restraint)/never allow yourself to eat more than you had planned (rigid restraint)
**Reward**	Reinforcement of achievement of specific behavior or outcome through reward contingent on the meeting of that target	Allowing ‘cheat’ or ‘treat’ meals after restricting for a certain amount of time
**Self‐monitoring**	Record specific behaviors or outcomes on regular basis	Use a pedometer to measure the amount of physical activity you do
**Stimulus control**	Alter personal environment such that it is more supportive of target behaviors (adapted from CALO‐RE)	Do not keep plates of food on table when eating
**Support: Buddying**	Perform target behaviors with another person	Exercise with a friend
**Support: Motivational**	Discussing, pledging, or revealing weight loss goals, plans, achievements, or challenges to others to bolster motivation	Discuss your weight loss goals with friends/family
**Support: Professional**	Seek help to manage weight from someone with specific expertise	Get support from a dedicated weight loss service or professional
**Weight management aids**	Use of and/or purchase of aids to achieve weight loss in any other manner (including, but not limited to, reducing energy intake and increasing energy output)	Use meal replacements to control weight

**Table 4:** Participant characteristics: field testing (completed first questionnaire, *n* = 167)

Characteristic	*n* (%)
**Gender**	138 (82.6%) Female
**Ethnicity**	151 (90.4%) White British/Irish 6 (3.6%) Other White 2 (1.2%) Black British 2 (1.2%) Other Black 2 (1.2%) South Asian 1 (0.6%) Other
**Education (highest level completed)**	7 (4.2%) No qualifications 57 (34.1%) O/A level or equivalent 85 (50.9%) University degree 18 (10.8%) Other/no information provided
**Age**	11 (6.6%) 20‐29 years old 25 (15.0%) 30‐39 54 (32.3%) 40‐49 53 (31.7%) 50‐59 21 (12.6%) 60‐69 3 (1.8%) 70+
**Weight**	Mean 96.8 kg (SD 21.0)

### Questionnaire

Think‐aloud interviews were conducted with 12 participants, with recruitment stopping when saturation was reached. Three men and nine women were interviewed; seven had a university degree, three were 30 to 39 years old, two were 40 to 49 years old, and seven were 50 to 59 years old. All participants had tried to lose weight multiple times. Think‐aloud testing led to the removal of some questions and the amending of others. The initial list of 200 questions was reduced to 117 questions (questions deleted where multiple questions existed for the same strategy). Approximately one third of the remaining questions were amended, mostly relating to specific words (e.g., weight control changed to weight management throughout). Multiple choice response options were determined during the think‐aloud phase after discussion with participants.

During field testing, 167 participants completed the Web‐based questionnaire at least once and 138 a second time. Participant characteristics are reported in Table 4. Of the 167 participants who answered the first survey, 137 were attempting to lose weight through changes to both their diet and physical activity, three with physical activity only, and 27 by diet only. All participants reporting using at least 22 (19%) of the strategies, with participants indicating use of 71 strategies on average (SD 17.7). The percentage using each strategy ranged from 7% (contingency contracting) to 92% (self‐weighing; setting personal weight loss goals).

The median bias index across all 117 questions was 0.03 (IQR: 0.015 to 0.06), ranging from 0 to 0.13 and indicating that the bias was low. The median prevalence index was 0.41 (IQR: 0.18 to 0.61), ranging from 0 to 0.9. The mean PABAK across all questions was 0.61 (SD = 0.15). Five questions had PABAK scores below 0.40 and were reconsidered and rephrased to improve clarity.

Ten questions were revisited because more than one participant indicated they found the question unclear (seven questions had two “unclears”; two questions had three “unclears”; one question had four “unclears”); these questions were also rephrased. No questions were removed. The final questionnaire can be found in Supporting Information Table S2.

## Discussion

The OxFAB taxonomy and questionnaire draw upon existing weight management resources, behavior change theories, and taxonomies. The questionnaire has been tested for face validity and revised where appropriate. Test–retest scores show it provides reliable measures of individual weight control strategies in most cases and further refinements are expected to enhance the reliability further. The taxonomy has been used to code interventions in a systematic review of self‐help interventions for weight loss, and the questionnaire is being used to track the behaviors individuals use in managing their weight as part of an observational cohort study.

The OxFAB taxonomy is distinct from other behavioral taxonomies due to its focus on the cognitive and behavioral strategies individuals use themselves, as opposed to those used by people delivering behavior change interventions. However, as would be expected, some elements of the OxFAB taxonomy map onto Michie et al.'s 93 item behavior change technique taxonomy 7. For example, in “action planning” (item 1.4 in Michie et al.'s 93 item taxonomy), a therapist may engage with a client to determine where, when, and how a person exercises. However, it is the individual who has to enact this, with various possible strategies. For example, the individual may schedule the activity, making reminders to assist memory. She/he may need to resist the impulses arising from a disinclination to exercise or use “impulse acceptance,” noticing the impulse but not acting on it. The Michie taxonomy describes the actions of the therapist in this scenario, whereas the OxFAB taxonomy and questionnaire are designed to describe the internal world of the person receiving the therapy, and the efforts they engage in to fulfil their plan.

There is no agreed methodology for taxonomy development 7. Future steps will include further discussions and collaborations with colleagues which may result in changes to the taxonomy, changes to descriptions of elements of the taxonomy, or both, which may in turn result in new questionnaire items after which formal reliability testing will be conducted. Accordingly, at this early stage of development, there are several limitations. This is only the first version, designed to generate discussion and to encourage consideration of the strategies from the perspective of the participant and not the therapist. There are legitimate considerations of completeness; though data saturation was reached in the initial stage of coding self‐management resources, and though think‐aloud interviews did not generate strategies not covered by the questionnaire, there may be some relatively less common strategies that are not currently included. Newly identified strategies can be incorporated in future iterations, as this initial version of the taxonomy is discussed and utilised by colleagues in the field and as the breadth of behavioral interventions expands. Currently, the OxFAB taxonomy and questionnaire deliberately exclude strategies outside of a person's own control and do not include those strategies which would be clinically contraindicated (e.g., purging) and their inclusion will hinge on feedback from users. Once a consensus has been reached that the breadth of strategies are adequately captured and the descriptions of domains are adequate to support accurate coding, the test–retest will be repeated and formal inter‐coder reliability will be performed.

Primarily, this taxonomy provides a starting point for discussion which will be enhanced as the tool is used. We may have missed some techniques that are used in some programs and welcome contact from others in the field to develop the OXFAB taxonomy and maximise its scope. We envisage that these tools can be used in a variety of ways. First, the OxFAB taxonomy can be used to encourage a systematic approach to the description of the cognitive and behavioral strategies advocated as part of interventions for weight management, such that the same strategy is described in the same terms, facilitating understanding and synthesis of research findings. Second, the questionnaire can be used to help identify the active ingredients of weight management attempts at the level of individual implementation. Third, both the taxonomy and questionnaire can be used to relate weight change to these behavioral components, and, subsequently, to prompt the development of new interventions designed to prompt the adoption of the most effective strategies.

Identifying effective personal strategies for weight management is important given the large number of adults trying to lose weight 2. Despite the growing popularity of online and mobile applications to support self‐directed weight management attempts 29, 30, 31, investigations into the content of self‐help resources has found that they are largely lacking in evidence‐based content 32, 33. Of those resources that are evidence based, evidence often comes from intensive interventions and it is conceivable that strategies recommended in these settings may not be as effective outside the context of these intensive interventions. This paucity of empirical evidence makes it difficult for individuals, or practitioners, to identify those strategies which have been shown to be effective. Identification of the personal strategies used by individuals during attempts at weight control and research into which strategies can increase the chances of successful weight loss and maintenance could be a key component of efforts to combat the obesity epidemic.

## Supporting information

Supporting Information
