# Supplementary material for: Development of tools to study personal weight control strategies: OxFAB taxonomy
Source: Obesity (Silver Spring). 2016 Jan 8;24(2):314–20. doi: 10.1002/oby.21341 (PMC4744943; doi:10.1002/oby.21341)
Supplement: Supplementary file 1 — Supporting Information [file OBY-24-314-s001.docx]

Development of tools to study personal weight control strategies: the Oxford Food and Activity Behaviours (OxFAB) taxonomy and questionnaire

# Supplementary information

Jamie Hartmann-Boyce, Paul Aveyard, Constantinos Koshiaris, Susan A Jebb; Nuffield Department of Primary Care Health Sciences, University of Oxford, Radcliffe Observatory Quarter, Oxford, OX2 6GG, UK.

## Table S1. Strategies grouped by domain

| **Strategy** | **Domain** | **Cross classification** |
| --- | --- | --- |
| Adjust the amount you eat in response to how much you have eaten/will eat | Energy compensation | food |
| Adjust the amount you eat in response to the amount you have exercised/will exercise | Energy compensation | food |
| Adjust the amount you exercise in response to how much you have eaten/will eat | Energy compensation | activity |
| Adjust the amount you exercise in response to how much you have excercised/will exercise | Energy compensation | activity |
| Set food intake targets | Goal setting | food |
| Set physical activity targets | Goal setting | activity |
| Set weight targets | Goal setting |  |
| Set one time targets | Goal setting |  |
| Set repeated time targets | Goal setting |  |
| Set achievable targets | Goal setting |  |
| Set body shape/size targets | Goal setting |  |
| Copying the diet behavior of others | Imitation (modelling) | food |
| Copying the physical activity behaviour of others | Imitation (modelling) | activity |
| Copying the weight management behaviour of others | Imitation (modelling) |  |
| Accept feelings of hunger | Impulse management: acceptance | food |
| Accept cravings to eat | Impulse management: acceptance | food |
| Accept uncomfortable aspects of physical activity | Impulse management: acceptance | activity |
| Ask yourself if you are hungry when you find yourself wanting to eat | Impulse management: awareness of motives | food |
| Ask yourself if you are full when part way through a meal | Impulse management: awareness of motives | food |
| Ask yourself why you feel like not being active | Impulse management: awareness of motives | activity |
| Find other activity to do when you feel like eating | Impulse management: distraction | food |
| When I'm uncomfortable whilst being active, I do something to distract myself from the sensation | Impulse management: distraction | activity |
| Hold off eating when you have a craving to eat | Impulse management: delay | food |
| Use physical devices, e.g gum shield after certain time to discourage further eating | Impulse management: substitution | food |
| Brush teeth to reduce desire to eat | Impulse management: substitution | food |
| Go to sleep if feeling hungry | Impulse management: substitution | food |
| Seek information on food content/ energy | Information seeking | food |
| Seek information on calories burned during exercise | Information seeking | activity |
| Seek information about how to manage weight | Information seeking |  |
| Record your motivation for losing weight | Motivation |  |
| Give yourself visual cue to remind yourself of motivation to lose weight (e.g. fat pictures/ slim pictures) | Motivation |  |
| Track your progress using chart/graph/body size simulator | Motivation |  |
| Concious appraisal of yourself to reinforce your actions (e.g. drawing on self-identity) | Motivation |  |
| Plan what you are going to eat (at home) | Planning content | food |
| Bring food for meals when eating away from home | Planning content | food |
| Plan what you are going to eat when you are eating out | Planning content | food |
| Prepare shopping list in advance | Planning content | food |
| Use recipes when cooking | Planning content | food |
| Carry healthy snacks for emergencies | Planning content | food |
| Incorporate physical activity into commute to work | Planning content | activity |
| Find types of physical activity that you enjoy | Planning content | activity |
| Create exercise plan | Planning content | activity |
| Do more housework/gardening as a way of increasing activity | Planning content | activity |
| Allow yourself to eat unlimited amounts of certain foods/ drinks | Regulation: allowances | food |
| Allowing 'cheat' or 'treat' meals, times or days after restricting for a certain amount of time | Regulation: allowances | food |
| Allowing physical activity free (or physical activity light) days after doing a certain amount of activity | Regulation: allowances | activity |
| Avoid certain kinds of food | Regulation: restrictions | food |
| Don't eat foods you don't particularly like | Regulation: restrictions | food |
| Don't eat foods you like | Regulation: restrictions | food |
| Think in advance about how to politely turn down food | Regulation: restrictions | food |
| Avoid eating in certain settings or at certain times | Regulation: restrictions | food |
| Avoid certain places where you might purchase food | Regulation: restrictions | food |
| Do not eat out of the packet | Regulation: restrictions | food |
| Go out less | Regulation: restrictions | food |
| Skip meals | Regulation: restrictions | food |
| Don't purchase certain types of food | Regulation: restrictions | food |
| Avoid eating at certain times of day | Regulation: restrictions | food |
| Purchase certain types of food | Regulation: rule setting | food |
| Leave the table after eating | Regulation: rule setting | food |
| Eat slowly | Regulation: rule setting | food |
| Leave food on the plate | Regulation: rule setting | food |
| Drink large glass of water or tea before meal to try and limit intake by feeling fuller quicker | Regulation: rule setting | food |
| Cut food into small pieces | Regulation: rule setting | food |
| Drink different type of alcohol/swap food for healthier option | Regulation: rule setting | food |
| Maximise potential for physical exertion when being active | Regulation: rule setting | activity |
| Accept some periods will exercise more dietary restraint than others (flexible) | Restraint | food |
| Rigid dietary restraint | Restraint | food |
| Accept some periods will be more rigid about meeting physical activity targets than others | Restraint | activity |
| Rigid physical activity restraint | Restraint | activity |
| Reward yourself for hitting targets | Reward |  |
| Set aside money at beginning and reward yourself by getting money back if you achieve your targets (contingency contracting) | Reward |  |
| Plan when you are going to eat | Scheduling of diet and activity | food |
| Eat at set times (mealtimes) | Scheduling of diet and activity | food |
| Schedule doing your food shopping at a time when you are unlikely to be hungry | Scheduling of diet and activity | food |
| Eat at meal times even when you are not hungry | Scheduling of diet and activity | food |
| Eat before going food shopping | Scheduling of diet and activity | food |
| Schedule physical activity | Scheduling of diet and activity | activity |
| Give up something else to make time for physical activity | Scheduling of diet and activity | activity |
| Exercise at times/in places where others are least likely to see you | Scheduling of diet and activity | activity |
| Go to bed at a set time each night | Scheduling of diet and activity |  |
| Measure the content of your food | Self-monitoring | food |
| Measure the amount of your food | Self-monitoring | food |
| Measure the amount of physical activity you do | Self-monitoring | activity |
| Monitor your fitness levels | Self-monitoring | activity |
| Weigh yourself | Self-monitoring |  |
| Measure your waist size/other parts of your body | Self-monitoring |  |
| Monitor tightness of clothes/ rings | Self-monitoring |  |
| Avoid going into a shop when hungry | Stimulus control | food |
| Use smaller plates or bowls | Stimulus control | food |
| Choose food packaged in individual portion | Stimulus control | food |
| Do not keep extra food on table | Stimulus control | food |
| Purchase smaller quantities | Stimulus control | food |
| Prepare healthy food in a way that makes it more appealing | Stimulus control | food |
| Store leftovers in portion controlled amounts | Stimulus control | food |
| Order small dish when eating out | Stimulus control | food |
| Keep unhealthy food/drinks out of the house | Stimulus control | food |
| Give yourself cues to increase physical activity | Stimulus control | activity |
| Give yourself cues to stand up/move when stationary | Stimulus control | activity |
| Diet with a friend/family member | Support: buddying | food |
| Exercise with a friend/family member | Support: buddying | physical activity |
| Get support from a someone you didn't know previously who is also trying to lose weight | Support: buddying |  |
| Enhance accountability to buddy | Support: buddying |  |
| Ask people around you not to offer you foods you are trying not to eat | Support: motivational | food |
| Ask people not to offer you foods you are trying not to eat | Support: motivational | food |
| Attend an exercise group | Support: motivational | activity |
| Get family/friend support | Support: motivational |  |
| Get support from people you live with | Support: motivational |  |
| Pledge/ agree to contract regarding your weight loss targets | Support: motivational |  |
| Commit to your weight loss plans in front of other people | Support: motivational |  |
| Seek help to help deal with feeling down, anxious or stressed | Support: professional help |  |
| Belong to a weight loss group | Support: professional help |  |
| See healthcare professional about weight loss | Support: professional help |  |
| Get support from a dedicated weight loss service or professional | Support: professional help |  |
| Use meal replacements to control weight | Weight management aids | food |
| Purchase/ use home exercise equipment | Weight management aids | activity |
| Purchase gym membership | Weight management aids | activity |
| Purchase/ use equipment to reduce sedentary time in office | Weight management aids | activity |

## Table S2. Questions in OxFAB, with questions grouped by domain

Note: answer options are: Most of the time; Sometimes; Never or hardly ever; Not relevant to me; Unclear

| **Question** | **Domain** |
| --- | --- |
| I try to balance how much I eat, so if eat a lot at one point I’ll make up for it by eating less at other points | Energy compensation |
| I adjust how much I eat depending on how much physical activity I’ve done or plan to do | Energy compensation |
| I adjust how much physical activity I do depending on how much I’ve eaten or am planning to eat later | Energy compensation |
| If I haven’t been very active, or know I’m going to be inactive for a while, I’ll exercise more at another point to balance it out | Energy compensation |
| I set myself goals for what or how much I’ll eat each day or each week | Goals |
| I set myself goals for how active I’ll be each day or each week | Goals |
| I know how I want to look and won’t give up losing weight until I am the size and shape I want to be | Goals |
| I have a goal weight in mind that I am working towards | Goals |
| I set goals which may be unrealistic | Goals |
| I want to achieve my weight loss targets within a set amount of time or by a certain date (e.g. I want to lose weight in time for my holiday) | Goals |
| I have a clear goal for the amount of weight I want to lose each week | Goals |
| I’ve decided to eat some particular foods and/or do certain types of physical activity in my attempt to lose weight because it worked for someone I know | Imitation |
| When I feel like eating something that doesn’t fit with my diet, I acknowledge the feeling but don’t act on it | Impulse management: Acceptance |
| When I feel uncomfortable or am in pain when I’m exercising, I accept it as I know it’s doing me good | Impulse management: Acceptance |
| If I feel like eating but am trying not to, I pause and ask myself if I’m hungry | Impulse management: Awareness of motives |
| Part way through eating a meal, I pause and ask myself if I’m full enough to stop | Impulse management: Awareness of motives |
| If I feel like being inactive, I ask myself why I feel that way | Impulse management: Awareness of motives |
| If I feel like eating but am trying not to, I find something else to do to distract myself | Impulse management: Distraction |
| When I have a craving for something I shouldn’t eat, I try to think about something else to take my mind off it | Impulse management: Distraction |
| If I feel like I want to stop exercising, I focus on something else to distract me so that I keep going | Impulse management: Distraction |
| If I feel like eating but am trying not to, I make myself wait a certain amount of time to see if the craving passes | Impulse management: Delay |
| If I feel like eating but am trying not to, I chew gum, drink something, or brush my teeth to stop me from doing so | Impulse management: Substitution |
| I try to go to sleep as a way to not eat when I am feeling hungry | Impulse management: Substitution |
| When I’m grocery shopping and items of food look similar, I make my choice based on the nutritional information on the food labels | Information |
| I use a book, website or app to look up the nutrition information and/or calorie content of the foods I eat | Information |
| I’ve looked up information on how many calories I burn doing physical activity | Information |
| I’ve looked up strategies, tips or plans for how to lose weight | Information |
| I check the nutritional information of the food I choose when eating out | Information |
| I use pictures of myself, other people, events, or places to remind me why I want to lose weight | Motivation |
| I look at clothes that I would like to wear but are currently too small to boost my motivation to lose weight | Motivation |
| I keep a note or a record to remind myself of my reasons for losing weight | Motivation |
| I keep myself motivated by reminding myself of the reasons I want to lose weight | Motivation |
| I use a chart, graph or diary to track my progress in losing weight | Motivation |
| I use an online program or app to look at my body shape and how it could change | Motivation |
| I draw on my values about what is important in life to motivate me to lose weight | Motivation |
| I plan my meals in advance to manage my weight | Planning content |
| When I’m away from home (e.g. at work), I take food with me so that I can stick to my eating plan | Planning content |
| If I’m going to eat out, I’ll plan what I’m going to eat before I get there to help me stick to my diet | Planning content |
| I plan my food shopping in advance to help me stick to my diet (e.g. use a shopping list) | Planning content |
| As part of sticking to my diet, I use particular recipes | Planning content |
| If I’m trying to get somewhere, I’ll walk or cycle some or all of the way as a way to help me manage my weight (e.g. cycling to work, walking to the shops instead of driving or taking the bus) | Planning content |
| I follow an exercise plan/routine | Planning content |
| I’ve taken to doing more chores at home/in the garden to help me get more exercise and lose weight | Planning content |
| I've chosen a type of physical activity I prefer in order to keep it up | Planning content |
| My diet allows me to I eat as much as I want of certain types of food and drinks | Regulation: Allowances |
| After I’ve been dieting for a certain amount of time, I’ll allow myself a food treat that isn’t part of my diet | Regulation: Allowances |
| I skip meals as a way to lose weight | Regulation: Restrictions |
| To help me manage what I eat, I try to avoid eating certain foods | Regulation: Restrictions |
| I try not to eat anywhere but at the table | Regulation: Restrictions |
| Part of my plan to lose weight is to eat only the foods I especially enjoy | Regulation: Restrictions |
| I avoid eating in certain settings or at certain times of day as a way to help me lose weight (e.g. in front of the television, on the go, late at night) | Regulation: Restrictions |
| There are specific shops or aisles in the supermarket that I avoid to help me stick to my diet | Regulation: Restrictions |
| To control how much I have, I put a certain amount of food on a plate or drink in a glass | Regulation: Restrictions |
| I choose not to go out because I am on a diet | Regulation: Restrictions |
| I think ahead about ways to turn down food when people offer it to me (e.g., before going to a party I think of reasons to give for turning down cake) | Regulation: Restrictions |
| I leave the table immediately after I’ve finished eating as a way to keep me from eating more | Regulation: Restrictions |
| I avoid eating some of my favourite foods because I’m worried if I start eating I may eat too much | Regulation: Restrictions |
| When I go food shopping, I try to only buy the types of food that I know I should be eating | Regulation: Rule setting |
| I slow down how quickly I eat in an effort to eat less | Regulation: Rule setting |
| I drink water or low calorie/low sugar drinks between alcoholic drinks to limit the amount of calories I consume | Regulation: Rule setting |
| I cut my food into small pieces to help me manage my eating | Regulation: Rule setting |
| I have water or a low-calorie drink or eat low-calorie food to help me eat less during meals | Regulation: Rule setting |
| When I am food shopping, there are certain foods I stay away from to help me stick to my diet | Regulation: Rule setting |
| I would swap one type of food or drink for another if I knew one would be better for my diet (e.g. lower fat or lower sugar versions of foods I normally eat) | Regulation: Rule setting |
| When I’m being active, I push myself to my limits | Regulation: Rule setting |
| I try to leave food on my plate at the end of a meal as a way to eat less | Regulation: Rule setting |
| I have a weight management plan, but I allow myself to be flexible about what I do depending on circumstances | Restraint |
| I’ve designated certain times/days as points where I don’t have to stick to my diet, in order to reward me for sticking to it at other points | Reward |
| I’ll reward myself for my sticking to my exercise goals by occasionally having days where I don’t do much physical activity | Reward |
| If I’ve reached one of my weight loss targets, I will reward myself with something that isn't food | Reward |
| I put aside money at the beginning of my weight loss attempt and only allow myself to have it back if I’ve hit my targets | Reward |
| I plan the times of day when I am going to eat to help me with my weight loss plans | Scheduling |
| I eat at mealtimes, even if I’m not hungry | Scheduling |
| I go to bed at a particular time each night to help me lose weight | Scheduling |
| I schedule physical activity into my week | Scheduling |
| I give up something else to make time for exercise (e.g. watching TV, socializing, leave work early) | Scheduling |
| I try to exercise at times or in places where other people are least likely to see me | Scheduling |
| I plan when I am going to do my food shopping to make sure it's not at a time when I am really hungry | Scheduling |
| I keep track of the calorie and/or nutritional content of the things I eat | Self-monitoring |
| I check the portion sizes of the things I eat | Self-monitoring |
| I keep track of the physical activity that I do | Self-monitoring |
| I measure my fitness levels (e.g. heart rate, how long it takes to do certain things, how much weight I can lift) | Self-monitoring |
| I keep track of my weight by weighing myself | Self-monitoring |
| I measure my waist (or other parts of my body) | Self-monitoring |
| I use the tightness of my clothes or rings to keep track of my weight | Self-monitoring |
| If I feel like eating but am trying not to, I avoid going into shops that sell food | Stimulus control |
| I use smaller plates, bowls or glasses when eating to help with my portion control | Stimulus control |
| I buy food pre-packaged in individual portions whenever I can | Stimulus control |
| As a way to keep me from overeating, I serve up food before bringing it to the table | Stimulus control |
| If I have leftovers, I’ll store them in portion controlled amounts to prevent me from eating too much later | Stimulus control |
| As a way to eat less, I share food when eating out | Stimulus control |
| I buy smaller amounts of certain foods to help me eat less | Stimulus control |
| To help me manage my weight, I order smaller dishes when eating at a restaurant (e.g. a starter or child size dish as a main) | Stimulus control |
| To avoid eating or drinking things that don’t fit with my diet, I don’t keep them at home | Stimulus control |
| I do something to prompt me to exercise (e.g. lay out my exercise clothes the night before) | Stimulus control |
| I have systems to remind me to be physically active during the day (e.g. reminders on my phone) | Stimulus control |
| I buy or prepare healthy foods in a way that makes me more likely to eat them (e.g. fruit salad instead of whole fruits, carrot batons instead of whole carrots) | Stimulus control |
| I am trying to lose weight alongside a friend/family member/my partner | Support: Buddying |
| I feel like I am part of a team with my friend(s)/partner/family member. We are losing weight together. | Support: Buddying |
| I have an online weight loss buddy | Support: Buddying |
| I exercise in a group and I think it is more enjoyable than exercising alone | Support: Motivational |
| I exercise in a group and it makes me work harder than I would do on my own | Support: Motivational |
| I’ve tried to get the people I live with to encourage my weight loss plans | Support: Motivational |
| I’ve tried to get my friends and family to support me in managing my weight | Support: Motivational |
| I warn people in advance  that I am dieting so that they don’t offer me foods I am trying not to eat | Support: Motivational |
| I told other people about my weight loss goals to help me stick to them | Support: Motivational |
| I’ve promised other people I'll lose a certain amount of weight | Support: Motivational |
| I belong to a group of people who are trying to lose weight together (for example, an online discussion forum, a group of colleagues all trying to lose weight) | Support: Motivational |
| I am losing weight with a friend/family member/my partner and I'm trying hard to lose more than them | Support: Motivational |
| I exercise with a group and when I don’t attend someone asks me why I wasn’t there | Support: Motivational |
| Feeling stressed, down, or anxious can make me break my diet so I sought help to tackle my feelings | Support: Professional |
| I’ve talked to a healthcare professional about managing my weight (e.g. doctor, nurse, dietitian, physiotherapist, psychologist) | Support: Professional |
| I use a weight loss service to help me manage my weight (for example, Weight Watchers, Slimming World, Lighter Life) | Support: Professional |
| I use meal replacements (e.g. shakes, diet bars, etc.) | Weight management aids |
| I use the gym as part of my weight loss efforts | Weight management aids |
| I use something at my desk that helps me stay more active when working (e.g. a standing desk) | Weight management aids |
| I exercise at home using my own equipment or DVDs | Weight management aids |

## Table S3. OxFAB items mapped onto Michie et al’s 93 item BCT taxonomy

| **Strategy** | **Domain** | **Relationship to 93 item BCT taxonomy (7)** |
| --- | --- | --- |
| Adjust the amount you eat in response to how much you have eaten/will eat | Energy compensation | No comparable techniques |
| Adjust the amount you eat in response to the amount you have exercised/will exercise | Energy compensation | No comparable techniques |
| Adjust the amount you exercise in response to how much you have eaten/will eat | Energy compensation | No comparable techniques |
| Adjust the amount you exercise in response to how much you have excercised/will exercise | Energy compensation | No comparable techniques (though broad conceptual overlap with broad conceptual overlap with BCT 1.1 Goal setting behaviour, BCT 2.3 Self-monitoring of behaviour, and BCT 1.5 Review of behavioural goals) |
| Set food intake targets | Goal setting | Grounded example (BCT technique 1.1 Goal setting (behaviour)) |
| Set physical activity targets | Goal setting | Grounded example (BCT technique 1.1 Goal setting (behaviour)) |
| Set weight targets | Goal setting | Grounded example (BCT technique 1.3 Goal setting (outcome)) |
| Set one time targets | Goal setting | No comparable techniques (though broad conceptual overlap with Category 1 Goals and planning) |
| Set repeated time targets | Goal setting | No comparable techniques (though broad conceptual overlap with Category 1 Goals and planning) |
| Set achievable targets | Goal setting | No comparable techniques (though broad conceptual overlap with Category 1 Goals and planning) |
| Set body shape/size targets | Goal setting | Grounded example (BCT technique 1.3 Goal setting (outcome)) |
| Copying the diet behavior of others | Imitation (modelling) | Related but not direct map (BCT technique 6.1 Demonstration of the behaviour; 6.2 Social comparison) |
| Copying the physical activity behaviour of others | Imitation (modelling) | Related but not direct map (BCT technique 6.1 Demonstration of the behaviour; 6.2 Social comparison) |
| Copying the weight management behaviour of others | Imitation (modelling) | Related but not direct map (BCT technique 6.1 Demonstration of the behaviour; 6.2 Social comparison) |
| Accept feelings of hunger | Impulse management: acceptance | No comparable techniques |
| Accept cravings to eat | Impulse management: acceptance | No comparable techniques |
| Accept uncomfortable aspects of physical activity | Impulse management: acceptance | No comparable techniques |
| Ask yourself if you are hungry when you find yourself wanting to eat | Impulse management: awareness of motives | No comparable techniques |
| Ask yourself if you are full when part way through a meal | Impulse management: awareness of motives | No comparable techniques |
| Ask yourself why you feel like not being active | Impulse management: awareness of motives | No comparable techniques |
| Find other activity to do when you feel like eating | Impulse management: distraction | Grounded example (BCT technique 12.4 Distraction) |
| Hold off eating when you have a craving to eat | Impulse management: delay | No comparable techniques |
| Use physical distraction, e.g gum shield after certain time to discourage further eating | Impulse management: substitution | No comparable techniques |
| Brush teeth to distract / reduce desire to eat | Impulse management: substitution | No comparable techniques |
| Go to sleep if feeling hungry | Impulse management: substitution | No comparable techniques |
| When I'm uncomfortable whilst being active, I do something to distract myself from the sensation | Impulse management: distraction | Grounded example (BCT technique 12.4 Distraction) |
| Seek information on food content/ energy | Information seeking | Related but no direct map (BCT Category 4 Shaping knowledge) |
| Seek information on calories burned during exercise | Information seeking | Related but no direct map (BCT Category 4 Shaping knowledge) |
| Seek information about how to manage weight | Information seeking | Direct map (BCT 4.1 Instruction on how to perform behaviour) |
| Record your motivation for losing weight | Motivation | No comparable techniques |
| Give yourself visual cue to remind yourself of motivation to lose weight (e.g. fat pictures/ slim pictures) | Motivation | No comparable techniques |
| Track your progress using chart/graph/body size simulator | Motivation | Related but no direct map (BCT 2.4 Self-monitoring of outcome(s) of behaviour) |
| Conscious appraisal of yourself to reinforce your actions (e.g. drawing on self-identity) | Motivation | Related but no direct map (BCT Category 13 Identity; BCT Category 15 Self-belief) |
| Plan what you are going to eat (at home) | Planning content | Grounded example (BCT 1.4 Action planning) |
| Bring food for meals when eating away from home | Planning content | Grounded example (BCT 12.1 Restructuring the physical environment) |
| Plan what you are going to eat when you are eating out | Planning content | Grounded example (BCT 1.4 Action planning) |
| Prepare shopping list in advance | Planning content | Grounded example (BCT 1.4 Action planning) |
| Use recipes when cooking | Planning content | Related but no direct map (BCT 1.4 Action planning) |
| Carry healthy snacks for emergencies | Planning content | Grounded example (BCT 12.1 Restructuring the physical environment) |
| Incorporate physical activity into commute to work | Planning content | Grounded example (1.4 Action planning) |
| Find types of physical activity that you enjoy | Planning content | No comparable techniques |
| Create exercise plan | Planning content | Grounded example (BCT 1.4 Action planning) |
| Do more housework/gardening as a way of increasing activity | Planning content | No comparable techniques |
| Allow yourself to eat unlimited amounts of certain foods/ drinks | Regulation: allowances | No comparable techniques |
| Allowing 'cheat' or 'treat' meals, times or days after restricting for a certain amount of time | Regulation: allowances | Grounded example (BCT 10.1 Material incentive (behaviour)) |
| Allowing physical activity free (or physical activity light) days after doing a certain amount of activity | Regulation: allowances | Related but no direct map (BCT 10.2 Material reward (behaviour)) |
| Avoid certain kinds of food | Regulation: restrictions | No comparable techniques |
| Don't eat foods you don't particularly like | Regulation: restrictions | No comparable techniques |
| Don't eat foods you like | Regulation: restrictions | No comparable techniques |
| Think in advance about how to politely turn down food | Regulation: restrictions | Related but no direct map (BCT 8.1 Behavioural practice/rehearsal) |
| Avoid eating in certain settings or at certain times | Regulation: restrictions | Related but no direct map (BCT 1.4 Action planning) |
| Avoid certain places where you might purchase food | Regulation: restrictions | Grounded example (BCT 12.3 Avoidance/reducing exposure to cues for the behaviour) |
| Do not eat out of the packet | Regulation: restrictions | No comparable techniques |
| Go out less | Regulation: restrictions | Grounded example (BCT 12.3 Avoidance/reducing exposure to cues for the behaviour) |
| Skip meals | Regulation: restrictions | No comparable techniques |
| Don't purchase certain types of food | Regulation: restrictions | Grounded example (BCT 12.3 Avoidance/reducing exposure to cues for the behaviour) |
| Avoid eating at certain times of day | Regulation: restrictions | No comparable techniques |
| Purchase certain types of food | Regulation: rule setting | Grounded example (BCT technique 12.5 Adding objects to the environment) |
| Leave the table after eating | Regulation: rule setting | Grounded example (BCT 12.3 Avoidance/reducing exposure to cues for the behaviour) |
| Eat slowly | Regulation: rule setting | No comparable techniques |
| Leave food on the plate | Regulation: rule setting | No comparable techniques |
| Drink large glass of water or tea before meal to try and limit intake by feeling fuller quicker | Regulation: rule setting | No comparable techniques |
| Cut food into small pieces | Regulation: rule setting | No comparable techniques |
| Drink different type of alcohol/swap food for healthier option | Regulation: rule setting | Grounded example (BCT 8.2 behaviour substitution) |
| Maximise potential for physical exertion when being active | Regulation: rule setting | No comparable techniques |
| Accept some periods will exercise more dietary restraint than others (flexible) | Restraint | No comparable techniques |
| Rigid dietary restraint | Restraint | No comparable techniques |
| Accept some periods will be more rigid about meeting physical activity targets than others | Restraint | No comparable techniques |
| Rigid physical activity restraint | Restraint | No comparable techniques |
| Reward yourself for hitting targets | Reward | Grounded example (BCT 10.9 Self-reward) |
| Set aside money at beginning and reward yourself by getting money back if you achieve your targets (contingency contracting) | Reward | Grounded example (BCT 10.9 Self-reward) |
| Plan when you are going to eat | Scheduling of diet and activity | Grounded example (BCT 1.4 Action planning) |
| Eat at set times (mealtimes) | Scheduling of diet and activity | No comparable techniques |
| Schedule doing your food shopping at a time when you are unlikely to be hungry | Scheduling of diet and activity | Grounded example (BCT 1.4 Action planning) |
| Eat at meal times even when you are not hungry | Scheduling of diet and activity | No comparable techniques |
| Eat before going food shopping | Scheduling of diet and activity | Grounded example (BCT 1.4 Action planning) |
| Schedule physical activity | Scheduling of diet and activity | Grounded example (BCT 1.4 Action planning) |
| Give up something else to make time for physical activity | Scheduling of diet and activity | Grounded example (BCT 8.2 behaviour substitution) |
| Exercise at times/in places where others are least likely to see you | Scheduling of diet and activity | Related but no direct map (BCT 1.4 Action planning) |
| Go to bed at a set time each night | Scheduling of diet and activity | Related but no direct map (BCT 1.4 Action planning) |
| Measure the content of your food | Self-monitoring | Grounded example (BCT 2.3 Self-monitoring of behaviour) |
| Measure the amount of your food | Self-monitoring | Grounded example (BCT 2.3 Self-monitoring of behaviour) |
| Measure the amount of physical activity you do | Self-monitoring | Grounded example (BCT 2.3 Self-monitoring of behaviour) |
| Monitor your fitness levels | Self-monitoring | Grounded example (BCT 2.4 Self-monitoring of outcome(s) of behaviour) |
| Weigh yourself | Self-monitoring | Grounded example (BCT 2.4 Self-monitoring of outcome(s) of behaviour) |
| Measure your waist size/other parts of your body | Self-monitoring | Grounded example (BCT 2.4 Self-monitoring of outcome(s) of behaviour) |
| Monitor tightness of clothes/ rings | Self-monitoring | Grounded example (BCT 2.4 Self-monitoring of outcome(s) of behaviour) |
| Avoid going into a shop when hungry | Stimulus control | Grounded example (BCT 12.3 Avoidance/reducing exposure to cues for the behaviour) |
| Use smaller plates or bowls | Stimulus control | Related but no direct map (BCT 12.1 Restructuring the physical environment) |
| Choose food packaged in individual portion | Stimulus control | Grounded example (BCT 12.1 Restructuring the physical environment) |
| Do not keep extra food on table | Stimulus control | Grounded example (BCT 12.1 Restructuring the physical environment) |
| Purchase smaller quantities | Stimulus control | Grounded example (BCT 12.1 Restructuring the physical environment) |
| Prepare healthy food in a way that makes it more appealing | Stimulus control | Related but no direct map (BCT 12.1 Restructuring the physical environment) |
| Store leftovers in portion controlled amounts | Stimulus control | Grounded example (BCT 12.1 Restructuring the physical environment) |
| Order small dish when eating out | Stimulus control | Related but no direct map (BCT 12.3 Avoidance/reducing exposure to cues for the behaviour) |
| Keep unhealthy food/drinks out of the house | Stimulus control | Grounded example (BCT 12.1 Restructuring the physical environment) |
| Give yourself cues to increase physical activity | Stimulus control | Grounded example (BCT 7.1 Prompts/cues) |
| Give yourself cues to stand up/move when stationary | Stimulus control | Grounded example (BCT 7.1 Prompts/cues) |
| Diet with a friend/family member | Support: buddying | Grounded example (BCT 3.1 Social support (unspecified/ BCT 3.3 Social support (emotional)) |
| Exercise with a friend/family member | Support: buddying | Grounded example (BCT Category 3 Social support) |
| Get support from a someone you didn't know previously who is also trying to lose weight | Support: buddying | Grounded example (BCT 3.3 Social support (emotional)) |
| Enhance accountability to buddy | Support: buddying | Related but no direct map (BCT Category 3 Social support) |
| Ask people around you not to offer you foods you are trying not to eat | Support: motivational | Related but no direct map (BCT Category 3 Social support; BCT 12.3 Avoidance/reducing exposure to cues for the behaviour) |
| Ask people not to offer you foods you are trying not to eat | Support: motivational | Related but no direct map (BCT 12.3 Avoidance/reducing exposure to cues for the behaviour; Category 3 Social support) |
| Attend an exercise group | Support: motivational | Grounded example (BCT Category 3 Social support/1.4 Action planning) |
| Get family/friend support | Support: motivational | Grounded example (BCT Category 3 Social support) |
| Get support from people you live with | Support: motivational | Grounded example (BCT Category 3 Social support) |
| Pledge/ agree to contract regarding your weight loss targets | Support: motivational | Direct map (BCT 1.8 Behavioural contract) |
| Commit to your weight loss plans in front of other people | Support: motivational | Direct map (BCT 1.9 Commitment) |
| Seek help to help deal with feeling down, anxious or stressed | Support: professional help | Related but no direct map (BCT 3.3 Social support (emotional) and 11.2 Reduce negative emotions) |
| Belong to a weight loss group | Support: professional help | Related but no direct map (BCT Category 3 Social support) |
| See healthcare professional about weight loss | Support: professional help | Related but no direct map (BCT Category 3 Social support) |
| Get support from a dedicated weight loss service or professional | Support: professional help | Related but no direct map (BCT Category 3 Social support) |
| Use meal replacements to control weight | Weight management aids | No comparable techniques |
| Purchase/ use home exercise equipment | Weight management aids | Grounded example (BCT 12.5 Adding objects to the environment) |
| Purchase gym membership | Weight management aids | No comparable techniques |
| Purchase/ use equipment to reduce sedentary time in office | Weight management aids | Grounded example (BCT 12.5 Adding objects to the environment) |
